# Supplementary material for: A Genome-Focused Investigation Reveals the Emergence of a Mycobacterium tuberculosis Strain Related to Multidrug-Resistant Tuberculosis in the Amazon Region of Brazil
Source: Microorganisms. 2024 Sep 2;12(9):1817. doi: 10.3390/microorganisms12091817 (PMC11434004; doi:10.3390/microorganisms12091817)
Supplement: Supplementary file 1 [file microorganisms-12-01817-s001.zip › Supplementary_v2/Supplementary.pdf]

Supplementary Table 1. List of publicly Mycobacterium tuberculosis lineage 4 genomes isolated in Brazil and used for comparative analysis.

Supplementary Table 2. Genotypic drug resistance profile of Mycobacterium tuberculosis SIT2517 using TB-profiler.

Supplementary Table 3. List of other variants not associated with drug resistance.

Supplementary Table 4. Single Nucleotide Variation distance matrix.

Supplementary Table 5. Manual pairwise comparison.

Supplementary Table 6. Genotyping profiling based on Spoligotyping, 24-loci MIRU-VNTR typing and Whole-genome sequencing

Supplementary Figure 2. Heat map demonstrating two principal chains of tuberculosis transmission within the SIT2517 genomes isolated in Pará, Brazil.

Supplementary Figure 1. Phylogenetic tree comprising the 15 *Mycobacterium tuberculosis* genome SIT2517 and the reference genomes obtained from other studies conducted in Brazil and representative lineages of the *M. tuberculosis* complex.
