# Supplementary figures and images for: A Genome-Focused Investigation Reveals the Emergence of a Mycobacterium tuberculosis Strain Related to Multidrug-Resistant Tuberculosis in the Amazon Region of Brazil
Source: Microorganisms. 2024 Sep 2;12(9):1817. doi: 10.3390/microorganisms12091817 (PMC11434004; doi:10.3390/microorganisms12091817)

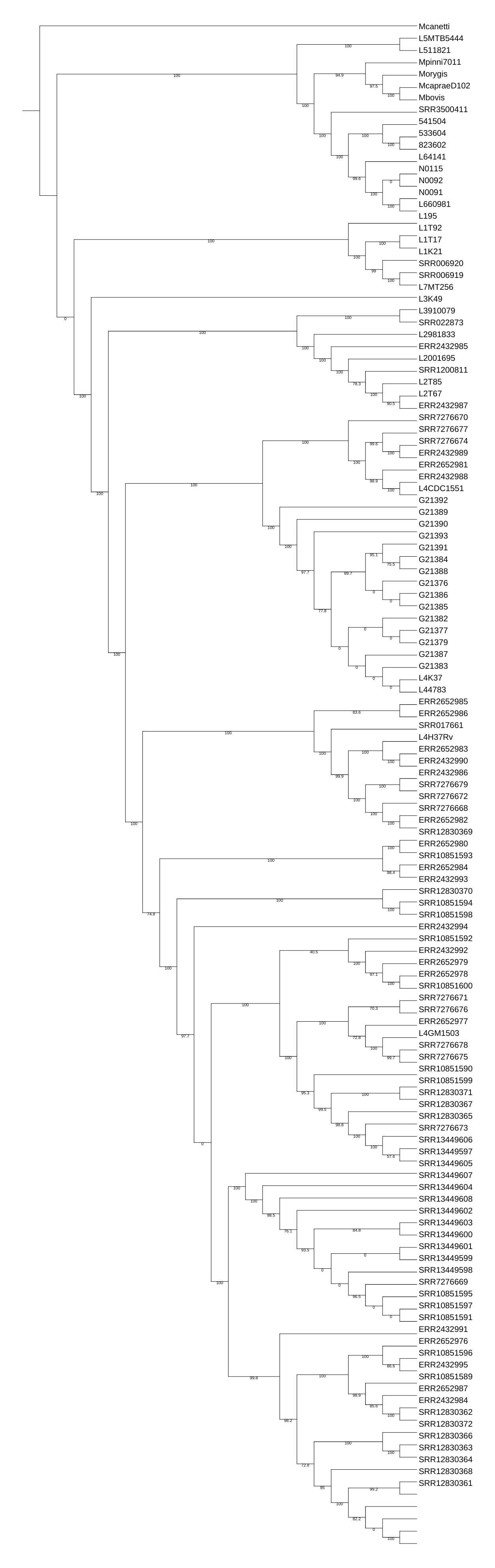

Supplement: Supplementary file 1 [file microorganisms-12-01817-s001.zip › Supplementary_v2/Supplementary Figure 1.png]

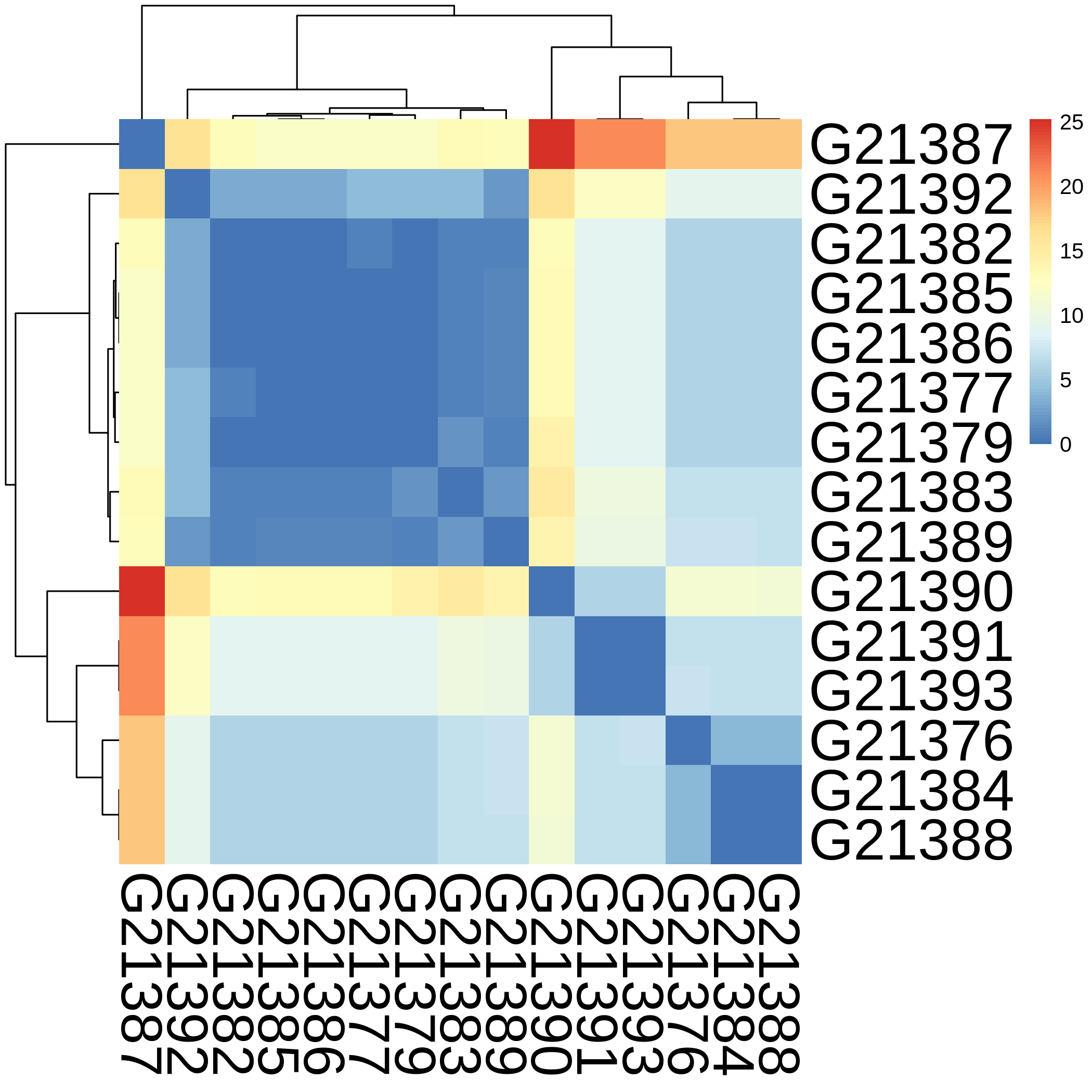

Supplement: Supplementary file 1 [file microorganisms-12-01817-s001.zip › Supplementary_v2/Supplementary Figure 2.png]
